# Structural and functional changes in the fungal community of plant detritus in an invaded Atlantic Forest

**DOI:** 10.1186/s12866-021-02431-8

**Published:** 2022-01-05

**Authors:** Jaqueline Bail, Jose Alejandro Morales Gomez, Giselle Cristina de Oliveira Vaz, Wagner Antonio Chiba de Castro, Rafaella Costa Bonugli-Santos

**Affiliations:** Federal University of Latin American Integration (UNILA), Institute Latin American of Nature and Life Sciences (ILACNV), Interdisciplinary Center of Life Sciences (CICV), 1000 Tarquínio Joslin dos Santos Av., Jardim Universitário, Foz do Iguaçu, PR 85870-901 Brazil

**Keywords:** Ascomycota, Basidiomycota, Iguaçu National Park, Litter decomposition, *Tradescantia zebrina*

## Abstract

**Background:**

Changes in the fungal community in the litter decomposition by invasive plants can negatively impact nutrient cycling in natural ecosystems. One still does not know the dimension of this hypothesis, but apparently, it is not despicable. This study evaluated the assemblage composition of fungi during litter decomposition in areas of Atlantic Forest invaded or not invaded by *Tradescantia zebrina* using Illumina MiSeq and metabarcoding analysis.

**Results:**

The invaded sample showed significantly higher richness and a difference in the species dominance than the invaded litter. Ascomycota was the first most abundant phylum in both areas. Even so, the dissimilarity between areas can be evidenced. The fungal from Basidiomycota were very representative in the non-invaded areas (ranged from an abundance of 43.29% in the non-invaded to 2.35% in the invaded sample). The genus *Lepiota* can indicate the primary functional group related to biomass degradation and showed the might difference about the invaded areas due to its essential reduction by the invader. In the invaded sample, there was a total absence of the endophyte-undefined saprotroph guild. Also, some genera not taxonomically characterized were eliminated in the invaded sample, revealing that the fungal biodiversity of areas has not yet been thoroughly characterized.

**Conclusions:**

Hence, makes impossible the real interpretation of the invasive plant impact, showing the importance of continuing research on fungal biodiversity. It is important to emphasize that the replacement of the native species by *T. zebrina* may be responsible for the elimination of fungal groups that have not yet been identified.

**Supplementary Information:**

The online version contains supplementary material available at 10.1186/s12866-021-02431-8.

## Background

The amount of soil organic carbon (SOC) in forest ecosystems is mostly driven by the interaction between plant litter (i.e. dry leaves and branches) [21] and decomposition by enzymes [5, 16, 35]. This interaction is the most valuable path of the biogeochemical cycle, that is, the flow of nutrients in the soil-plant-soil system [19, 49, 53].

Saprotrophic fungi are the most abundant organic source of biomass on Earth. They are responsible for functions such as the natural degradation of cellulose, hemicellulose, and recalcitrant substances, such as lignin, due to their use of a wide variety of enzymes [36, 69]. They actively participate in the processes of decomposition and nutrient cycling as they degrade dead organic matter, including the most abundant components, such as wood, litter, and soil organic matter [3, 37]. They are represented by the Basidiomycota and Ascomycota and classified according to their different rot strategies. Mild rot (ascomycetes) can play important and underestimated roles, while white and brown rot are most easily recognizable [15, 31].

Since plant organic matter influences both soil activity and microbial biomass, changes in vegetation (i.e. density of invasive plant species) are capable of significantly altering microbial communities and can also affect belowground diversity because of shifts in mycorrhizal composition and their competitiveness with saprotrophic fungi and, consequently, the ecosystem as a whole [18, 33, 34]. Dominant invasive plants can change the fixation of SOC through litter contribution and differential decomposition [6, 46]. The greater availability of degraded litter promoted by the invaders causes an increase in the metabolic activity of certain soil microorganisms and, consequently, the flow of carbon from the soil to the atmosphere [18]. However, the impact on the fungal community is not yet fully known, especially for uncultivated diversity.

The herbaceous *Tradescantia zebrina* is originally from Mexico and northern Central American countries, was brought to Brazil for ornamental purposes [38], and cited in several lists of invasive species [23, 70]. It has a high capacity for adaptation to different Brazilian biomes, with records of invasion in the Cerrado and Atlantic Forest ([38]. It multiplies quickly at any time of the year, establishing large biomass in invaded areas. Also, it is considered a strong competitor, affecting the diversity of species in forest fragments [38].

This study aimed to evaluate the fungal community during litter decomposition in Atlantic Forest areas invaded and not invaded by *T. zebrina*. Using metabarcoding and FUNGuild analysis, we expected to detect changes in the assemblage composition and functional diversity of fungi under invasion.

## Results

### Metabarcoding analyses of fungi from litter in the invaded and non-invaded areas of the INP

A total set with 113,390 forward reads was generated for the two samples. After quality processing (Filtering, denoising, and chimera removal), a final set was obtained with 64,389 sequences (supplementary material, Table 1). These sequences were grouped into 361 ASV’s. After low-frequency filtering and denoised, 117 ASV’s were kept. According to the rarefaction curve, both two samples showed that sufficient sequencing depth was obtained in our study (supplementary material, Fig. 1).

### Diversity and richness analysis of fungi from litter in the invaded and non-invaded areas of the INP

The α diversity and richness indexes were investigated in each sample at the level of ASV. Table 1 shows a significant difference from samples (ANOVA and Duncan’s new multiple range test *p*-values < 0.05). The invaded sample showed significantly higher richness and uniformity than the non-invaded litter and a difference in the species dominance. The dissimilarity between areas can be evidenced through the β-diversity (non-invaded area = 1; invaded = 0,1613).

**Table 1:** Biodiversity and richness estimators (Shannon index and Simpson index) of fungi, calculated at the taxonomic rank of ASVs, from litter in invaded and non-invaded areas of the INP after 100 days of incubation in a litter bag system

Samples	ASVs	Chao1	Shannon	Simpson
**Non-invaded**	74	73	2757	0,8467
**Invaded**	91	90	3523	0,9385

### Composition of fungi communities from litter in the invaded and non-invaded areas of the INP

**Fig. 1:**
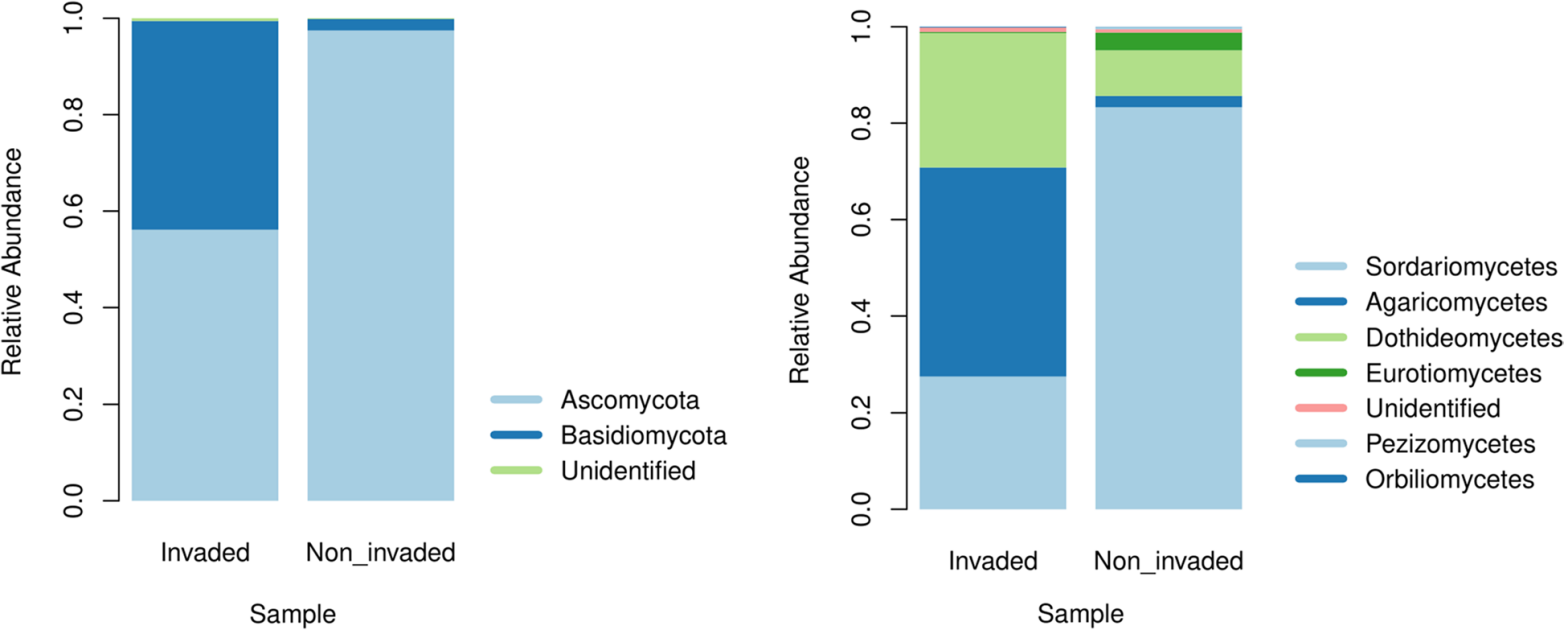
Relative frequency of phyla and classes of fungi from litter in the invaded and non-invaded areas of the INP after 100 days of incubation in a litter bag system

Taxonomic classifications and ASV annotations were assigned to taxa at seven taxonomic ranks, representing two fungal phyla (Fig. 1). As expected, the Ascomycota was the most abundant phylum found in both samples, representing 56.14% abundance in the non-invaded sample and 97.48% in the invaded sample. The Basidiomycota phylum ranged from an abundance of 43.29% in the non-invaded to 2.35% in the invaded sample. The Sordariomycetes class was more abundant in the invaded than the non-invaded sample (83.30 to 27.52%, respectively). In the non-invaded sample, 70% of ASVs as distributed among Agaricomycetes and Dothideomycetes (supplementary material, Fig. 2, Venn’s diagrams illustrate both the magnitude of the microbiota identified and those taxonomical levels that are unique to one sampling site and those that are shared).

**Fig. 2:**
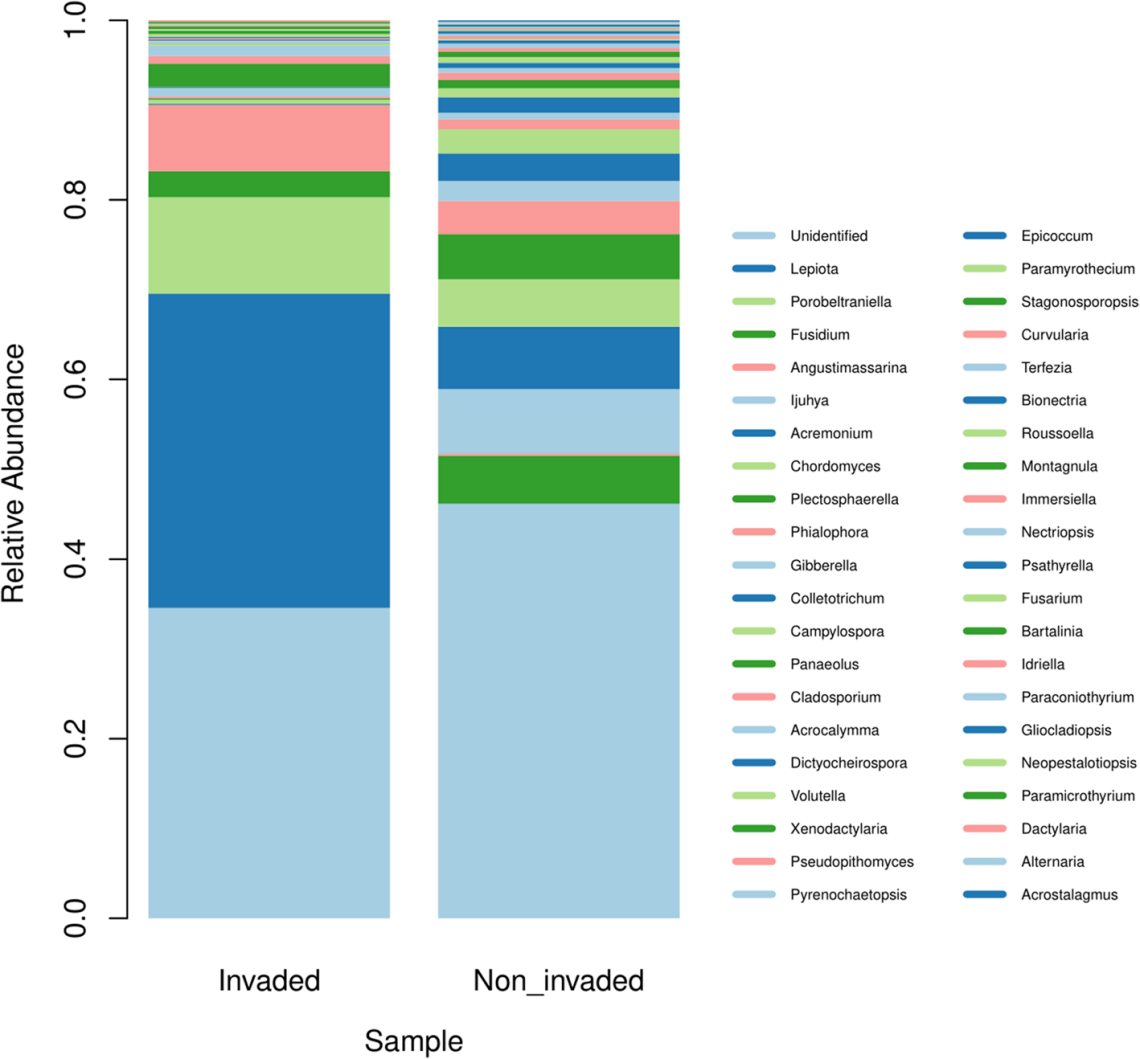
Relative abundances, at genus level, of fungi from litter in the invaded and non-invaded areas of the INP after 100 days of incubation in a litter bag system

Overall, some high abundance genera were not identified, 34.58% from the non-invaded sample, and 46.14% from the invaded sample (Fig. 2). Of the genera identified in both samples, *Lepiota* (Basidiomycota) were the most abundant with 34.97% (only 0.01% in the invaded sample), followed by *Porobeltraniella (*Ascomycota) with 10.75% (only observed in the invaded sample), *Angustimassarina* (Ascomycota) with 7.38% (especially in the invaded sample), *Ijuhya* (Ascomycota) with 7.19% (only observed non-invaded sample), and *Acremonium* (Ascomycota) with 6.94%.

The abundances of these genera differed when comparing non-invaded and invaded samples (GLMM, z-value = 24.63, *p* < 0,001). In the non-invaded sample, the genera mentioned above obtained these frequencies: *Lepiota* 34.97%*, Porobeltraniella* 10.75%, *Angustimassarina 7.38%, Acremonium* 0.12%, and the genus *Ijuhya* was not found. While in the invaded sample the genus *Lepiota* obtained 0.01%, *Porobeltraniella* was not found, the genus *Angustimassarina* obtained 0.23% frequency*, Ijuhya 7.19%,* and *Acremonium* 6.94%.

As shown in Fig. 2, four genera were exclusive to the non-invaded sample: *Porobeltraniella* (10.75%), *Panaeolus* (2.59%), *Montagnula* (0.35%), and *Paramicrothyrium* (0.21%). In addition, nine genera were found only in the invaded sample, such as *Ijuhya* (7.19%), *Paramyrothecium* (0.64%), *Stagonosporopsis* (0.62%), *Terfezia* (0.48%), *Immersiella* (0.32%), *Psathyrella* (0.30%), *Gliocladiopsis* (0.25%) and *Acrostalagmus* and *Alternaria* with (0.16%). It is noteworthy that the *Lepiota* genus obtained only 0.01% of frequency in the invaded sample, representing an abrupt reduction.

**Fig. 3:**
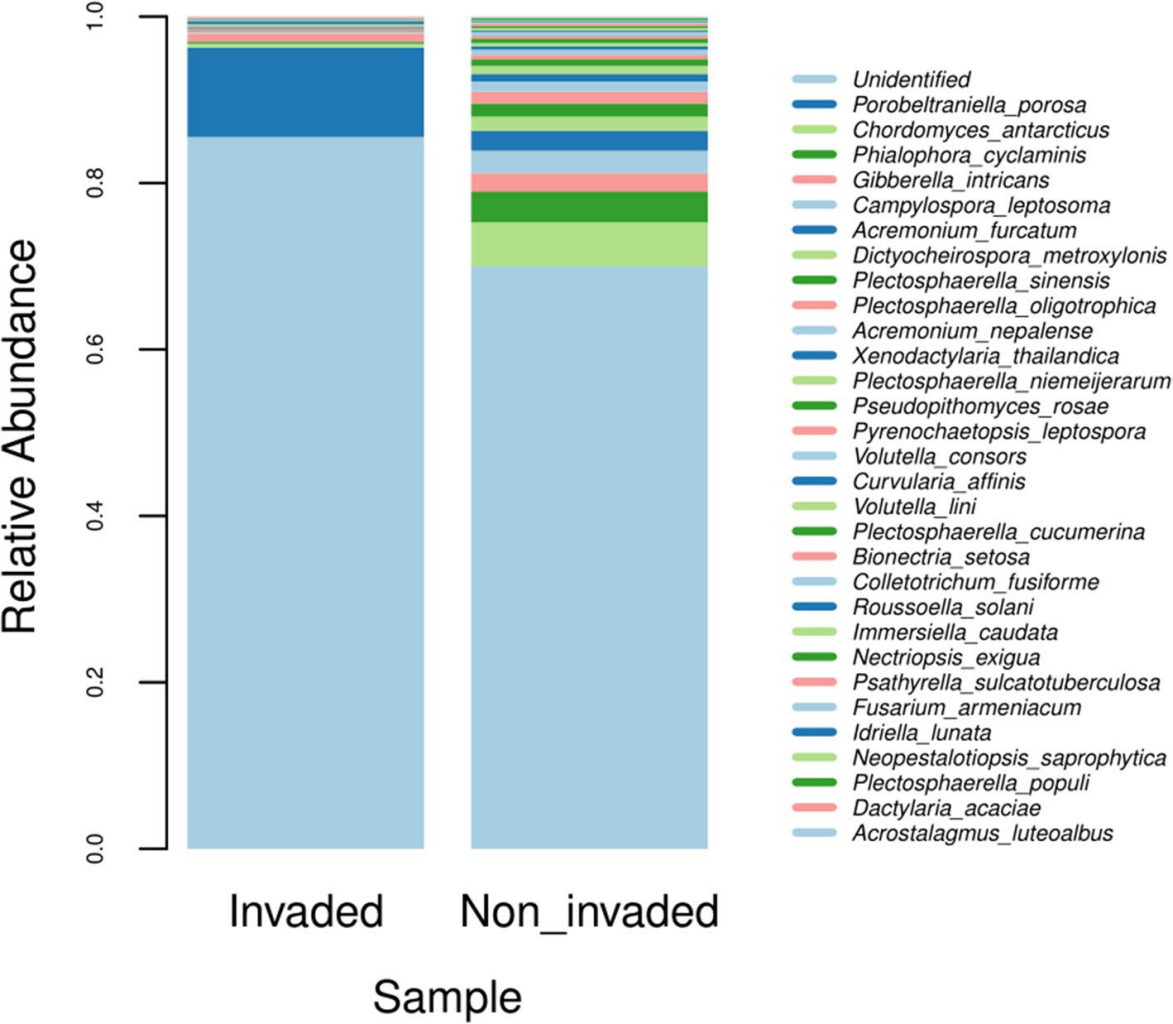
Relative abundances, at specie level, of fungi from litter in the invaded and non-invaded areas of the INP after 100 days of incubation in a litter bag system

Overall, some species of high abundance were not identified, 85.54% from the non-invaded sample and, 69.99% from the invaded sample (Fig. 3). Of the species identified in both samples, *Porobeltraniella porosa* were the most abundant with 10.75% (found only in the non-invaded sample), followed by *Chordomyces antarcticus* 5.29%, *Phialophora cyclaminis* 3.70%, *Campylospora leptosome* 2.70%, and *Acremonium furcatum* with 2.35%.

### Fungal functional profile

**Fig. 4:**
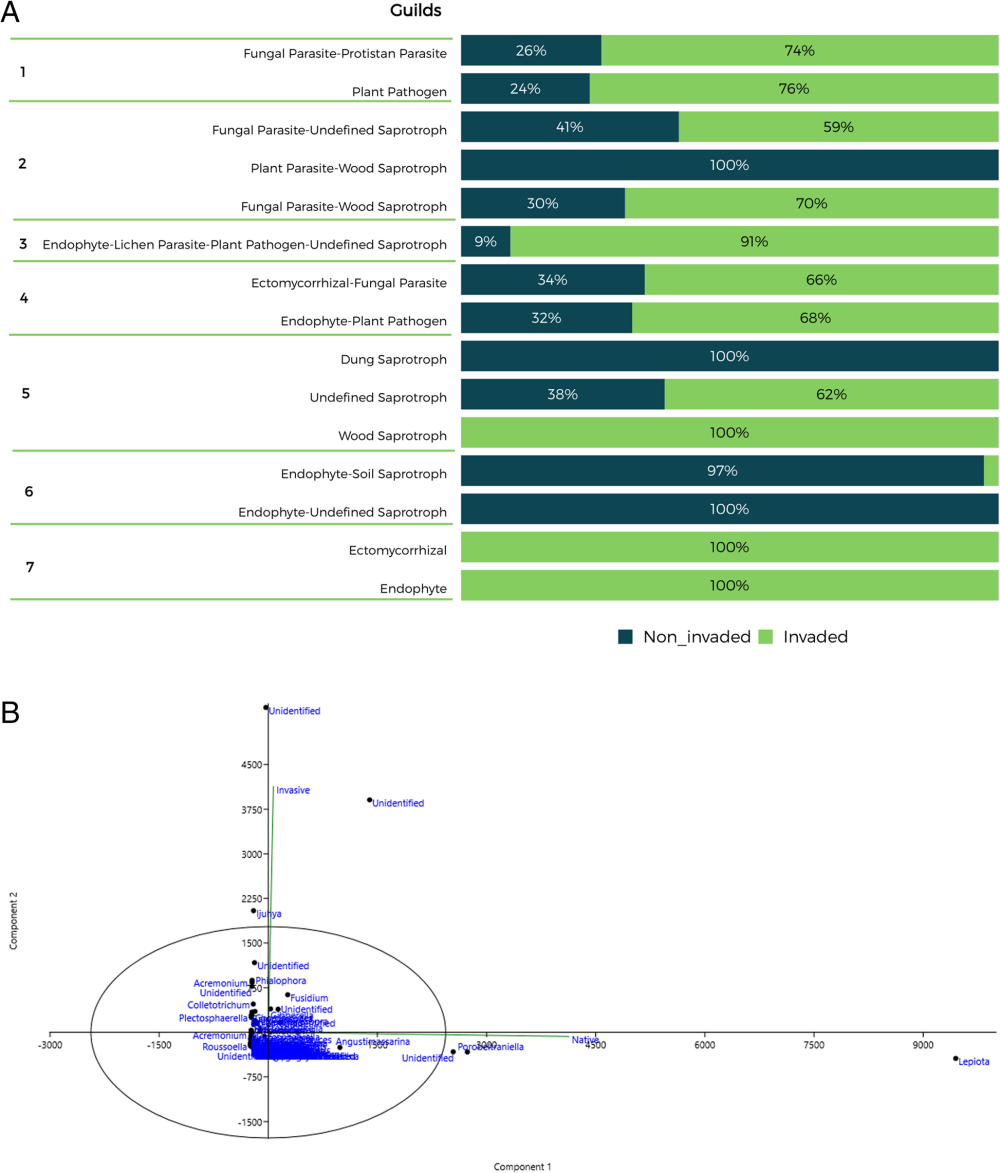
**A** Relative abundance of fungal functional ASVs inferred by FUNGuild from litter in the invaded and non-invaded areas of the INP after 100 days of incubation in a litter bag system. Guilds assignments were classified in seven trophic modes: 1) Pathotroph 2) Pathotroph-Saprotroph 3) Pathotroph-Saprotroph-Symbiotroph 4) Pathotroph-Symbiotroph 5) Saprotroph 6) Saprotroph-Symbiotroph 7) Symbiotroph; **B** Principal component analysis (PCA) of functional ASVs inferred by FUNGuild

FUNGuild analysis was conducted to predict microbial functional profiling. Our results showed that fungal ASVs were mainly distributed among the three guilds: endophytes, plant pathogens, and wood saprotrophs. Noteworthy is the endophyte-plant pathogen guild with 45.71%, the non-invaded sample with 14.55%, and the invaded sample with 31.16% (Fig. 4). Furthermore, the abundance of saprotrophs overlapped the other classes with a 58.35% total abundance of classified ASVs. Overall, there was a predominance of undefined saprotrophs (generalist saprotrophic fungi), whose decomposition targets are indefinite. This group may be due to a general lack of knowledge regarding fungal diversity [62, 67]. The analysis highlighted that the Dung Saprotroph and Endophyte-Undefined Saprotroph guilds were practically exclusive to the non-invaded sample.

## Discussion

The α-diversity and the dissimilarity between areas, referred to as β-diversity, could reflect two different ecological situations. It could reflect the loss of species from one site compared to the other. Alternatively, each site could be different because they host none of the species present at the other site. In practice, both events occur at the same time and were evidenced in the study [45].

The results indicated a significant increase in fungi abundance in the invaded sample due to *Tradescantia zebrina* biomass’s low lignin concentration characteristic [52]. Low lignin content facilitates the presence of non-ligninolytic saprophytic fungi, suggesting a functional redundancy profile. In our study, this profile was from the greater availability of necromass promoted by saprophytes fungi from invaded regions. These fungi can promote and increase metabolic activity in this area [18, 69]. Also, since the samples were obtained after 100 days of incubation, the functional redundancy found can be explained as being due to the samples undergoing the plant decomposition stage, where fungal diversity is increased, which means many species with similar functions. Conversely, at the beginning of the decomposition process, less diverse communities contain fungi with unique functions in the decomposition process [58]. The FUNGuild analysis reaffirmed the functional redundancy profile, and the absence of synergisms, especially for the invaded sample, since the richness of the majority guilds found was more prominent than the native sample.

In the non-invaded sample, 99% of the taxa were allocated to the phyla Ascomycota and Basidiomycota (56.14 and 43.29%, respectively). In the invaded sample, there was a precipitate reduction in the presence of basidiomycetes (only 2.35% of abundance). This result is consistent with Kerfahi et al. [29], a study addressing tropical forests that have been altered by logging and cleared for agriculture. According to these authors, the lower abundance of Basidiomycota might occur due to the relative lack of thick woody debris in the litter biomass, resulting in low lignin residues. So, the adverse effects on the fungal community caused by *T. zebrina* invasion would be comparable to deforestation.

The *T. zebrina* cell wall’s characteristics, especially the low lignin content [52], explain the predominance of the phylum Ascomycota. Eichlerová et al. [12] showed low activity of the enzymes involved with plant decomposition, including laccase, in the phylum Ascomycota’s saprophytic fungi compared to the phylum Basidiomycota. The comparative study of CAZymes (enzymes that act on carbohydrates from plant biomass) in the fungal genomes from classic types of fungal decomposers (white rot, brown rot, and soft rot) showed that basidiomycetes have the high potential ligninolytic capacity [27, 69].

Changes in the diversity of the invaded sample can directly result in a functional shift. As in the increase of endophytic fungi by improving Sordariomycetes class, also known as powdery mildew, found predominantly in the invaded sample. Still, ligninolytic enzymes such as laccase, vital for the decomposition of lignocellulose, can occur in endophytes and saprophytes fungi. In addition, also shown the isolate’s ability to switch between endophytic and saprophytic lifestyles, reinforcing the view of them as potential decomposers playing ecological roles in nutrient cycling and ecosystem stability.

Therefore, despite the almost complete absence of CAZymes from basidiomycetes in the invaded sample, the possibility that these enzymes are active cannot be ruled out. For example, a comparison of enzymatic activities in ascomycetes from *Quercus petraea* litter shows that basidiomycetes produced significantly less esterase, lipase, acid phosphatase, b-glucosidase, n-acetylglucosaminidase, a-mannosidase, and, especially, laccase [51], which indicates that internal colonizers (endophytes) may be more important in the production of these enzymes. This activity is crucial for lignin degradation since lignin-degrading enzymes depend on these enzymatic supports, such as oxidases, reductases, and esterases, to trigger hydrogen peroxide supply [28]. As shown by Zhang et al. [69] the transcriptomic pattern points to the mechanistic distinction between white and brown rot fungi. This metabolic distinction is revealed in the “signatures” left on the wood, where the loss of carbohydrates outweighs the loss of lignin during brown rot and contrariwise during white rot.

The most representative genus in the study concerning litter decomposition and CAZyme activity was *Lepiota* (order Agaricales, class Agaricaceae). This genus recorded the highest total abundance in the samples, which was significantly higher in the non-invaded sample (34.97%) than in the invaded sample (0.01%). It is essential to highlight that this group that belongs to the *Lepiota* genus has not yet been characterized at the species level; that is, the replacement of the native species by *T. zebrina* may be responsible for the elimination of fungal groups that have not yet been identified. *Lepiota* grows on soils with relatively high pH in high temperatures and rapidly decomposes litter [17]. They are also found in soils with high heavy metal content [26] which are common characteristics of the native soil of INP. It is known that this genus expresses genes encoding CAZymes, such as the enzyme pyranose dehydrogenase, where the Pdh1 gene of the glucose-methanol-choline superfamily of oxidoreductases can oxidize a wide variety of carbohydrates that arise during the degradation of lignocellulose [13, 14, 30, 47, 50, 56]. The genus *Lepiota* can indicate the primary functional group in our study related to biomass degradation and showed the might difference about the invaded areas due to its essential reduction by the invader.

The genera with the highest total abundance in the invaded sample, *Ijuhya,* and *Acremonium*, belong to the order Hypocreales (class Sordariomycetes), which includes biotrophic fungi, mainly plant and, insect pathogens, mycoparasites, endophytes, and various saprotrophic species [54, 66, 68]. Also, they are known to exhibit cellulolytic, lignocellulolytic, and hydrolytic activity [2, 55, 59], which explains their contribution to decomposition, nutrient cycling, and carbon fixation.

Some genera have not been identified (34.58% from the non-invaded sample and 46.14% from the invaded sample), revealing that the fungal biodiversity of INP has not yet been completely characterized. This absence of identification may be resulting in a lack of clarity regarding functional activity in the non-invaded sample and the effect of removing these groups on the invaded sample.

In general, there was dominance from saprotrophs fungi, which are represented by Basidiomycota and Ascomycota. Saprotrophs degrade dead organic matter, including the most abundant components, such as wood, litter, and soil organic matter. These fungi recycle large amounts of carbon and other nutrients, as they have several enzymes that make them capable of degrading all the structural components of dead plants [3, 37, 69]. The lignocellulolytic saprotrophs may also be active in several guilds, as similar enzymes are shared among the litter and dead wood saprotrophs [12, 32, 42, 60]. Wood saprotrophs are the most abundant organic source of biomass on Earth. They are responsible for functions such as the natural degradation of cellulose, hemicellulose, and recalcitrant substances such as lignin to their use of a wide variety of enzymes [36, 69].

On the other hand, we also detect a total absence of the Endophyte-Undefined Saprotroph and Dung Saprotroph guilds in the invaded sample. Saprotroph fungi were most extensively studied in agriculturally significant grass hosts. These systemic endophytes were shown to produce toxic alkaloids and even alter plant community assembly. Furthermore, these systemic endophytes were reported to cause slower litter decomposition rates in terrestrial systems [65]. In recent work, Wolfe and Ballhorn [65] analyze the findings of 67 studies on foliar endophytes’ roles in litter decomposition and their effects on decomposition rates. The authors suggest that host plant and endophyte interactions can significantly influence litter decomposition rates and should be considered when interpreting both terrestrial and in-stream litter decomposition experiments. Based on this report, we can indicate that this group’s exclusion in the invaded sample can trigger acute biological effects. More studies specifically exploring foliar endophyte effects on litter decomposition are needed.

The reduction of Dung-inhabiting fungi also deserves attention. Dung-inhabiting fungi play an essential ecological role in decomposing and recycling nutrients from animal dung. They produce many bioactive secondary metabolites and have a potent enzymatic arsenal able to utilize even complex molecules [48]. Consequently, these metabolites have biological functions that, in their absence, impact soil quality and biogeochemical cycles.

It is important to emphasize that microbial communities are not only characterized by the number and composition of taxa. But, too by the ecological associations among microbiome members. The community’s taxa do not necessarily determine the complexity of microbial networks and the relation with function. The number of associations that those taxa share also represents an important variable. Wu and Cox [66] provided new insights into the lifestyle transitions of fungi and, in particular, emphasized that relatively few essential genes are needed for fungi to adapt to new environments. The next frontier is to test empirically that the microbiome changes are essential for microbial communities to support ecosystem functioning [61]. The results obtained here will be indispensable for understanding that.

## Conclusions

The two environments studied was detected the wide taxonomic diversity and a wide range of functional guilds. The dissimilarity between areas was evidenced. Some groups were lost entirely in the litter invaded by *T. zebrina*. The massive reduction in the Basidiomycota group in the invaded sample showed the fungal diversity decreased, especially those producing ligninolytic enzymes. The change in the guilds (guild not detected in the invaded litter) can result in biological effects on soil quality and biogeochemical cycles.

Metabarcoding allowed us to confirm the cases already documented in other studies with other methodologies where invasive plants are more productive than native species and influence the local litter’s microbial diversity, where the diversity found was twice as high as that of native biomass. As a novelty, our study indicated structural and functional change. But also, it was possible to suggest that unknown taxonomic groups were eliminated in the invasion. What is the function? What impact this change has on the stability of the ecosystem? Knowing who these fungi are, our results indicate a series of issues that need to be evaluated from our data, of great environmental importance.

## Methods

### Study area

The Iguaçu National Park (INP) is a protected area in the western region of Parana State, Southern Brazil (25° 05′ to 25° 41′ S and 53° 40′ to 54° 38′ W) occupying 185,262.5 ha [24]. The climate in the region is subtropical Cfa (Köppen’s classification) with hot summers [1]. Mean annual precipitation varies from 1600 to 1800 mm, and mean annual temperature ranges from 22 °C to 23 °C [22]. INP constitutes the largest remaining area of Atlantic Forest in Southern Brazil, which comprises semideciduous seasonal forests, mixed ombrophilous forests, and early successional alluvial forests. A large number of plant and animal species native to Brazil occur in the Park, including some threatened species [24]. Although it has protected status, INP is not exempt from natural and anthropogenic impacts, such as agricultural pressure, hunting, and changes to the native flora. *Tradescantia zebrina* ex Bosse, a monocotyledonous plant from the Commelinaceae, likely native to Mexico and Northern Central America [38], has invaded several regions of the INP. Currently widely distributed in the world as an introduced species [20], it is amongst the most common invasive alien species in protected areas of Brazil.

Ten study areas of the PNI were selected, five having been invaded by *T. zebrina* and five not invaded. The invaded areas are currently highly invaded by *T. zebrina* (over 80% of dominance) and belong to distinct invasions (i.e. they formed discontinuous invaded sites representing different populations). All the areas were at least 500 m distant from one another.

### Collection of samples

Living fragments of (1) *T. zebrina* in the five invaded areas and (2) herbaceous and native trees/shrubs in the five non-invaded areas were collected. The native flora samples were collected in due time, with manual removal of fragments of herbs and leaves of trees and shrubs, as well as some dry senescent branches with clear signs of abscission. The invasive and native fragments were dried separately in an oven at 55 ± 5 °C until constant weight and added in litter bags. The ten experimental areas, being five non-invaded and five invaded ones, are located between 20 and 30 m from the edge of the forest, at least 500 m apart. In each area, six litterbags were placed (225 cm^2^ and 5 g of plant litter): three litterbags with dry fragments from *T. zebrina* (invader litter) and three with dry fragments from the native flora (native litter). The litter bags in each area were deposited alternately, 30 cm apart, and monitored over 4 months. On the 100th day of follow-up, the litter bags were collected and taken to the laboratory. Litters from invaded areas (both invader and native origin) were mixed to produce a composite sample (the invaded sample), the litters from non-invaded areas were treated the same way (the non-invaded sample).

### DNA extraction, ITS amplification and Illumina sequencing

The samples were kept at − 80 °C until total DNA extraction. From each composite sample, 2 g were macerated in liquid nitrogen and extracted using a DNeasy Plant Mini Kit (Qiagen Inc., Valencia, CA, USA). DNA concentration was quantified with a Qubit dsDNA BR Assay Kit (Thermo Fisher Scientific). PCR reaction and sequencing were performed by BPI Biotechnology Company. The PCR reaction produced a final volume of 20 μL, containing 10 μL of GoTaq Colorless Master Mix 2x (Promega, USA) 0.3 μM of ITS-86F primer (5′- TCGTCGGCAGCGTCAGATGTGTATAAGAGACAGGTGAATCATCGAATCTTTGAA – 3′) [57], 0.3 μM of ITS-4R primer (5′ – GTCTCGTGGGCTCGGAGATGTGTATAAGAGACAGTCCTCCGCTTATTGATATGC – 3′), 1 μL of genomic DNA (100 ng/ μL), and ultrapure water. Amplification consisted of initial denaturation at 95 °C for 5 min, followed by 35 cycles of denaturation at 95 °C for 30 s, annealing at 56 °C for 40 s, extension at 72 °C for 1 min, and a final extension at 72 °C for 5 min. The ITS1 was amplified in a multiplexing PCR using a set of barcoded primers (ITS-86F and ITS-4R) conducted in a Veriti™ thermal cycler (Applied Biosystems). The reaction was confirmed in 2% agarose gel stained with UniSafe Dye 0.03% (v/v), 400 bp (amplicon size). The amplicons were purified using an Agencourt AMPure XP magnetic bead (Beckman Coulter) and an indexing reaction by Nextera XT Index kit (Illumina). The libraries were submitted to purification steps using Agencourt AMPure XP magnetic beads (Beckman Coulter) and quantification by PCR in Real-Time using KAPA-KK4824 Library Quantification Kit (Illumina/Universal) and sequenced using the Illumina MiSeq new generation sequencing system (Illumina® Sequencing) with MiSeq Reagent Kit V2 Nano 500 cycles - 2 × 250 bp reading.

### Definition of amplicon sequence variants (ASVs) and community analysis

Overall quality inspection, filtering, denoising, and chimera removal to collect amplicon sequence variants (ASV’s) was made thorough DADA2 ITS Pipeline Workflow version 1.8 [10] implemented at R software [25], following recommendations from the author. To get better results and taxonomic resolution of the fungal soil community, only forward reads were kept for downstream analyses as advised by Pauvert et al. [43].

The “Filter And Trim” function was used for Quality filtering with options that included removal of primers, ambiguous bases (N), and sequences with less than 100 bp and an expected error (MaxEE) higher than 2. After quality filtering, an error model for sequencing was built with “learnErrors” function and forward reads were dereplicated with “derep” function to collapse repeated sequences. These sequences were denoised to get a set of ASV’s, and filtered for chimera detection with “removeBimeraDenovo” function. Finally, to account for potential contaminations and index bleed, a table with ASV’s counts and a set of denoised sequences, were exported. ASVs with a low frequency (less than 50) were excluded from table counts and denoised sequences.

To perform sequencing classification and taxonomic assignment, a machine learning classification approach implemented at Qiime2 [8] was applied. Table with ASV’s counts, denoised sequences, and UNITE database reference version 8.2 [41] for dynamic classification (97–99% similarity) designed for Qiime, were imported into Qiime2 [8] thorough a qiime artifacts. First, a classifier was created and trained on database reference using “qiime feature-classifier” method [7]. Once the classifier was trained, this one was used to classify and assign taxonomic labels to ASV’s with “Qiime feature-classifier classify-sklearn” method [44]. Classified sequences with assigned taxonomy and ASV’s table counts, were analyzed and plotted with R software [25], using Phyloseq [39] and ggplot2 [64] packages.

The α diversity matrices were used to analyze fungal diversity [63]. The rarefaction curve was performed with a sampling depth of 500 reads to capture the diversity present in samples with high read counts, but low enough to include the majority of samples. The α diversity of the areas was assessed using the Shannon diversity index (H′) and Simpson’s dominance index (C) [9]. A one-way analysis of variance (ANOVA) followed by a Duncan’s new multiple range test was applied to verify the significance of differences in alpha diversity among the matrices [4]. To characterize the dissimilarities between samples (β-diversity), the Bray–Curtis index was calculated. To predict the functional profiles of the fungal community, ecological guilds were annotated and parsed using the FUNGuild database, only confidence scores of ‘Probable’ and ‘Highly Probable’ were used to perform analysis [40].

We assessed the effect of the invasion on the specific abundance of the fungal community using generalized linear mixed models (GLMM). In this model, fixed effects included each taxon as a categorical factor, with 117 levels and interactions with environmental treatment effect (invaded or non-invaded samples). The random-effects structure included a random intercept for each resampling (as each taxon), nested within the environmental treatment [11]. The model was built using the R software package lme4, assuming a negative binomial distribution. Significance values for fixed effect were obtained either in the software package ImerTest using the Satterthwaite approximation for degrees of freedom.

## Supplementary Information

**Additional file 1: Supplementary Figure 1.** Rarefaction curve from litter in the invaded and non-invaded areas of the INP after 100 days of incubation in a litter bag system. **Supplementary Figure 2.** Venn’s diagrams from litter in the invaded and non-invaded areas of the INP after 100 days of incubation in a litter bag system. Illustrates both the magnitude of the mycrobiota identified and those taxonomical levels that are unique to one sampling site and those that are shared. A) Phylum; B) Class; C) Order; D) Family; E) Genus; F) Species. **Supplementary Table 1.** DNA sequencing quality processing from from litter in invaded and non-invaded areas of the INP after 100 days of incubation in a litter bag system.

## Data Availability

Raw data is deposited National Center for Biotechnology Information (NCBI): SRR13485875 and SRR13485876.

## Abbreviations

ANOVA: Analysis of variance
AVS: Amplicon sequence variant
CAZymes: Carbohydrate active enzymes
GLMM: Generalized linear mixed models
INP: Iguaçu National Park
SOC: Soil organic carbon
