# Supplementary material for: Structural and functional changes in the fungal community of plant detritus in an invaded Atlantic Forest
Source: BMC Microbiol. 2022 Jan 5;22:10. doi: 10.1186/s12866-021-02431-8 (PMC8729104; doi:10.1186/s12866-021-02431-8)
Supplement: Supplementary file 1 — Additional file 1: Supplementary Figure 1. Rarefaction curve from litter in the invaded and non-invaded areas of the INP after 100 days of incubation in a litter bag system. Supplementary Figure 2. Venn’s diagrams from litter in the invaded and non-invaded areas of the INP after 100 days of incubation in a litter bag system. Illustrates both the magnitude of the mycrobiota identified and those taxonomical levels that are unique to one sampling site and those that are shared. A) Phylum; B) Class; C) Order; D) Family; E) Genus; F) Species. Supplementary Table 1. DNA sequencing quality processing from from litter in invaded and non-invaded areas of the INP after 100 days of incubation in a litter bag system. [file 12866_2021_2431_MOESM1_ESM.docx]

Supplementary Material

# Supplementary Figures

**Supplementary Figure 1.** Rarefaction curve from litter in the invaded and non-invaded areas of the INP after 100 days of incubation in a litter bag system.


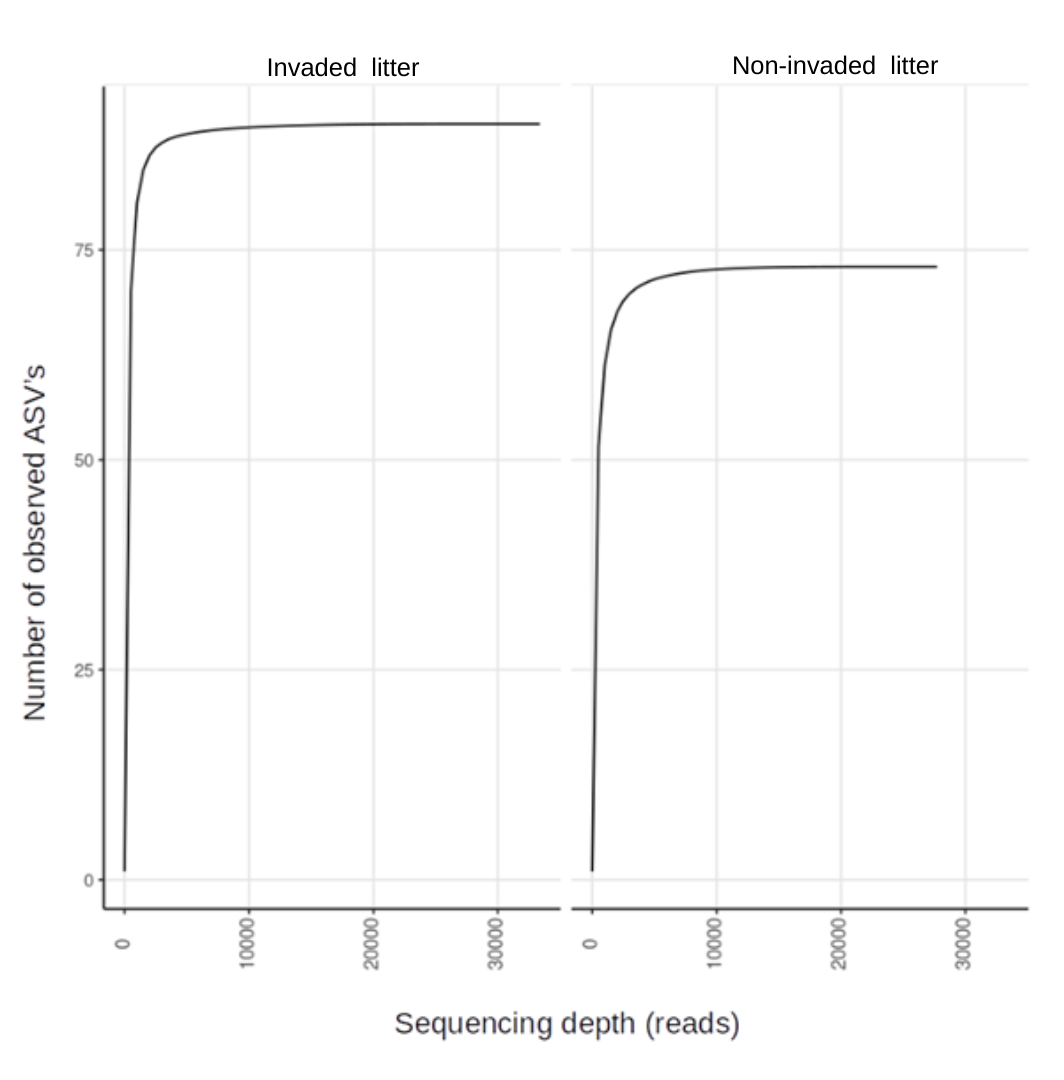


**Supplementary Figure 2.** Venn’s diagrams from litter in the invaded and non-invaded areas of the INP after 100 days of incubation in a litter bag system. Illustrates both the magnitude of the mycrobiota identified and those taxonomical levels that are unique to one sampling site and those that are shared. A) Phylum; B) Class; C) Order; D) Family; E) Genus; F) Species.


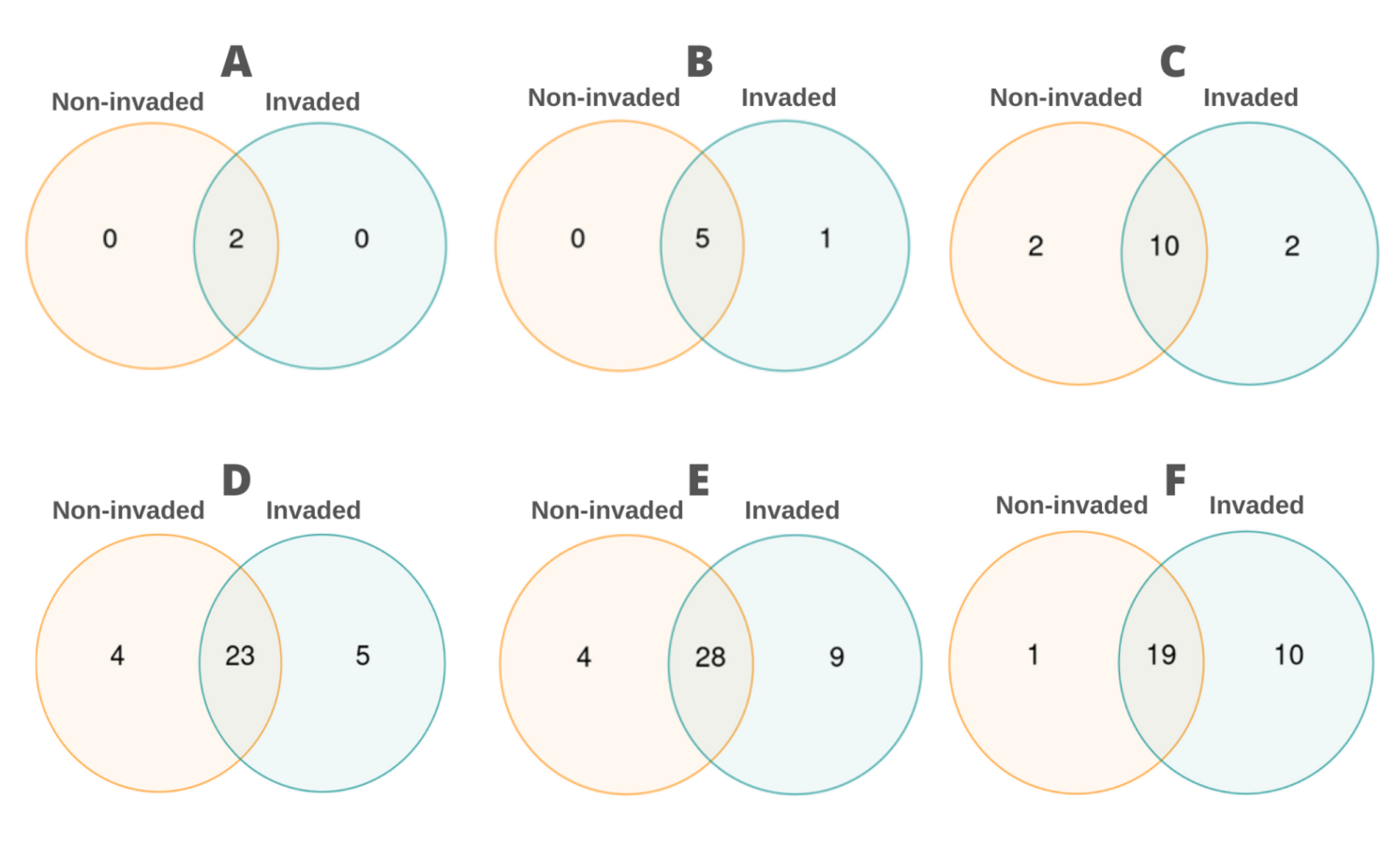


# Supplementary Tables

**Supplementary Table 1.** DNA sequencing quality processing from from litter in invaded and non-invaded areas of the INP after 100 days of incubation in a litter bag system.

| **Sample** | **Initial**  **sequences** | **Filtered**  **sequences** | **Denoised**  **sequences** | **Non chimeric**  **sequences** | **ASV after sequencing processing** | **ASV after low frequency filtering** |
| --- | --- | --- | --- | --- | --- | --- |
| **Non-invaded** | **48430** | **30749** | **29618** | **29199** | **361** | **117** |
| **Invasive** | **64960** | **37757** | **36946** | **35199** |  |  |
